# N,N‐Dimethyltryptamine (DMT) Acutely Exposed to Mouse Ventral Tegmental Area I_h_‐Negative Neurons Alters the Firing Rate and Conductance in a Sex‐Dependent Manner

**DOI:** 10.1111/jnc.70463

**Published:** 2026-05-18

**Authors:** Jannik Nicklas Eliasen, Amir Rezagholizadeh, Helene Påbøl Jacobsen, Kristi A. Kohlmeier

**Affiliations:** ^1^ Drug Design and Pharmacology, Faculty of Health and Medical Sciences University of Copenhagen Copenhagen Denmark; ^2^ Sino‐Danish Center (SDC) University of Chinese Academy of Sciences Beijing China

**Keywords:** dimethyltryptamine, electrophysiology, psychedelic, sex‐differences

## Abstract

Depression and substance use disorder (SUD) affect millions globally and unfortunately, while established treatments exist, they are not always adequate. Psychedelics have emerged as a promising avenue for development of more effective pharmacological interventions. Dimethyltryptamine (DMT), a classic psychedelic, has shown anti‐depressive and anti‐addictive properties in preclinical studies, observational evaluations, and limited controlled trials; however, the exact mechanism(s) of action(s) are still unknown. In the present ex vivo study, we provide the first evaluation of the electrophysiological effects of acute DMT exposure at two concentrations in male and female I_h_‐negative neurons of the mouse ventral tegmental area (VTA). The VTA plays a central role in regulating emotion and motivated behavior, and I_h_‐negative neurons, which are putatively inhibitory, shape VTA output to downstream targets. At the lower concentration, DMT (500 nM) did not exert any effect on evaluated electrophysiological properties of female and male VTA I_h_‐negative neurons. At the highest concentration (90 μM), DMT elicited a conductance change at subthreshold potentials and an increase in action potential firing; however, these actions were seen only in female VTA. At the higher concentration, DMT increased cytosolic calcium levels in both sexes. In conclusion, DMT has actions in both male and female I_h_‐negative VTA neurons, but the alterations in firing and membrane conductance observed in females indicate activation of mechanisms beyond the calcium changes seen in males. Taken together, these findings highlight the importance of translational research to connect the cellular effects of DMT with its potential long‐term therapeutic outcomes in humans, while accounting for variables such as sex and dose‐dependent responses.

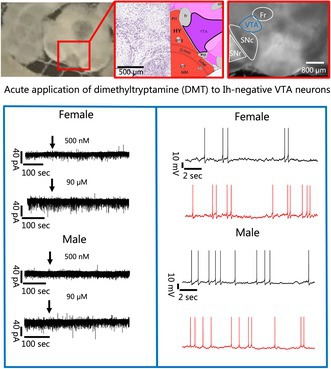

## Graphical abstract

Depression and substance use disorder are difficult to treat, prompting interest in psychedelics like DMT, though its neural mechanisms remain unclear. This ex vivo study examined acute DMT effects on electrophysiology in male and female I_h_‐negative neurons of the mouse ventral tegmental area (VTA). Low‐dose DMT (500 nM) had no effect, whereas a higher dose (90 μM) increased cytosolic calcium in both sexes but altered membrane conductance and firing only in females. These findings show sex‐ and dose‐dependent cellular actions of DMT and suggest mechanisms beyond calcium signaling, highlighting the need for translational research linking cellular effects to therapy.

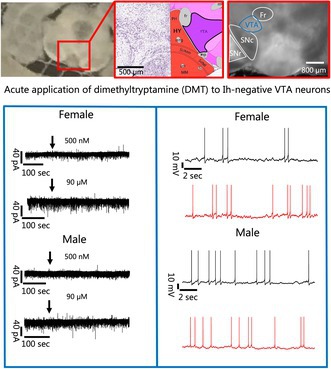

**Abbreviations**
ACSF: artificial cerebrospinal fluid
AHP: afterhyperpolarization
AP: action potential
DMT: dimethyltryptamine
F: the average fluorescence
F_0_
: fluorescence baseline
Fr: fasciculus retroflexus
I‐V: current–voltage
LSD: lysergic acid diethylamide
MAO: mono amine oxidase
NMRI: naval medical research institute
PFC: prefrontal cortex
PND: postnatal day
ROI: region of interest
sEPSCs: spontaneous excitatory postsynaptic currents
SNc: substantia nigra pars compacta
SNr: substantia nigra pars reticulata
ST: superthreshold
SUD: substance use disorder
TrKB: tropomyosin receptor kinase B
VTA: ventral tegmental area

## 1 Introduction

Mental health disorders such as clinical depression and substance use disorders (SUD) have become increasingly prevalent worldwide. Depression affects over 300 million individuals globally. Traditional treatments for depression, including selective serotonin reuptake inhibitors, often have delayed onset to effect (weeks to months) and limited efficacy, with up to 30%–50% of depressed patients showing inadequate response (Rush et al. 2006). Globally, nearly 400 million individuals used drugs in 2022, and only 1 in 11 of those suffering from SUD received treatment. When pharmacologically managed, SUD is associated with relapse rates that remain unacceptably high. For example, 80% of tobacco users relapse after one year (Medioni et al. 2005) even when pharmacological agents are coupled with interventions. Depression and SUD represent major public health challenges and, as current pharmacological options have limited efficacy, evaluation of novel approaches to treatment is warranted.

Although political pressure led to a decades‐long ban on research with psychedelics and their classification as having no medical value and high potential for abuse, psychedelic compounds that target the 5‐HT_2A_ receptor have experienced a resurgence of scientific interest as potential therapeutics. This interest stems in part from an emergence of pre‐clinical studies and clinical trials indicating that psychedelics could be potential treatments for mental health disorders. Among the psychedelics under consideration is N, N‐dimethyltryptamine (DMT) – a potent, highly psychedelic, and short‐acting tryptamine endogenously present in the human body (Dean et al. 2019). DMT is also contained in ayahuasca, a plant brew used for centuries in traditional medicine, which has recently been shown in anecdotal reports, observational studies, and randomized placebo‐controlled trials to exert promising anti‐addictive and anti‐depressant effects (Daldegan‐Bueno et al. 2022; Palhano‐Fontes et al. 2019; van Oorsouw et al. 2022; Osório et al. 2015; Thomas et al. 2013).

While the therapeutic mechanisms underlying DMT's anti‐addictive and antidepressant effects remain unclear, its psychedelic properties are known to be primarily mediated by 5‐HT_2A_ receptor activation, as evidenced by suppression of head‐twitch behavior in receptor‐knockout mice (González‐Maeso et al. 2007) and through pharmacological blockade (Moliner et al. 2023). Motivated by growing clinical evidence and anecdotal reports suggesting beneficial effects of DMT and ayahuasca in treating mood and substance use disorders, neurophysiological studies ‐ including the present work ‐ are increasingly focused on identifying the receptor and circuit‐level mechanisms that might account for these therapeutic outcomes. Given their importance in affective disorders, cortical and limbic regions, especially the prefrontal cortex (PFC), amygdala, and hippocampus have been the central focus of most research into DMT's neurophysiological effects. Functional imaging studies show that DMT increases global brain connectivity, with fMRI revealing enhanced amygdala–orbitofrontal coupling, modulation of emotion and affective cognitive circuits (Soares et al. 2024) and deactivation of the hippocampus which is linked to alterations in self‐perception and changes in self, time, and space which are actions believed to contribute to therapeutic effects (Timmermann et al. 2023). These macroscopic enhancements in network connectivity are paralleled by cellular‐level changes, including changes in synaptic strength and dendritic spine density in the cortex and hippocampus, which have been attributed to 5‐HT_2A_ actions, and involvement of the tropomyosin receptor kinase B (TrKB) receptor (Moliner et al. 2023; Ly et al. 2018; Vargas et al. 2023; Ilchibaeva et al. 2022; Jones et al. 2009), although other receptors are also likely involved (Morales‐Garcia et al. 2020; Fontanilla et al. 2009; Barker 2018; Carbonaro and Gatch 2016; Bunzow et al. 2001). Collectively, while much remains to be determined regarding the specific mechanisms of action at a cellular level, these findings highlight DMT's capacity to engage multiple neuroplasticity mechanisms across cortical and hippocampal systems, which likely contribute to the alterations in functional connectivity seen at the network level.

Understandably, the majority of studies of the neurophysiological actions of DMT have investigated effects in the cortex and hippocampus. However, the ventral tegmental area (VTA) plays a key role in the neurobiology of depression and SUD (Nair‐Roberts et al. 2008; Swanson 1982), and electrophysiological studies examining VTA neuronal responses to DMT remain absent. The majority of neurons in the VTA release dopamine, and dysregulation of VTA dopamine neuronal firing is evident in both SUD and depression. The firing and output of VTA dopaminergic neurons are critically shaped by the activity of I_h_‐negative GABAergic neurons also present in this nucleus. Further, GABA VTA neurons exhibit marked plasticity during exposure to drugs of abuse and withdrawal, suggesting their modulation could play a role in altering dopaminergic output, which is importantly involved in affective disorders (Eshel et al. 2015; Nugent et al. 2007; Bouarab et al. 2019; Brown et al. 2012; Li et al. 2019; Stamatakis et al. 2013). Accordingly, actions of DMT on GABAergic VTA neurons could play a role in therapeutic actions; however, whether DMT has actions on this cell phenotype is unknown. Thus, we have evaluated the electrophysiological response of putative GABAergic VTA neurons in female and male mouse brain slices to two concentrations of DMT. To this end, we recorded from I_h_‐negative neurons as the absence of I_h_‐current is often associated with the GABA phenotype in VTA neurons. We analyzed membrane currents, conductance, resting membrane potential, action potential (AP) firing rate, rheobase, afterhyperpolarization (AHP) amplitude/duration, and intracellular calcium transients before and after DMT exposure. Our data suggest that DMT exerts differential effects on the activity of I_h_‐negative neurons in the VTA, suggesting sex‐ and concentration‐based actions on the regulation of dopamine output to downstream regions implicated in mood and reward regulation.

## 2 Methods

Outbred Naval Medical Research Institute (NMRI) mice (Crl: NMRI; Scanbur, Karlslunde, Denmark) of both sexes were obtained from Charles River Laboratories and bred in‐house; pups aged postnatal day (PND) 14–25 were used for the present study. In total, data from 66 animals were included in the study (37 females and 29 males). The animal studies were approved by the Animal Welfare Committee appointed by the Danish Ministry of Justice (Permit number: 2020‐15‐0201‐00441). The European Communities Council Directive of 22nd of September 2010 (2010/63/EU) and the Danish legislation regulating the use of experimental animals were followed concerning housing and experimentation. NMRI mice (Scanbur; Karlslunde, Denmark) were housed under conditions noted to be effective in reducing stress and anxiety in experimental mice (Gouveia and Hurst 2013; Gouveia and Hurst 2019; Gurfein et al. 2012; Moons et al. 2004). Specifically, the animal facility was maintained at a constant temperature (22°C ± 2°C) and humidity (36%–58%) and illuminated under a 12‐h light/dark cycle (Lights on from 07:00 AM). Mice were housed in an open cage system (Cage size: 18″ × 12″ × 6″) with *ad libitum* access to food and water. All pups were housed with the mother until PND 21, where females and males were weaned and separated with a maximum of five same‐sex mice per cage. Cages were enriched with hiding places. Mice were transferred through handling tunnels to the transport box. Data were analyzed 24–48 h post experimentation to ensure only the necessary number of animals were sacrificed to evaluate the hypothesis. The use of pre‐ and peri‐adolescent mice controlled for effects due to hormonal modulations such as the estrous cycle, which does not occur until vaginal opening at ~26 PND, which is followed by the onset of the menstrual cycle (Caligioni 2009; Nelson et al. 1982). No blinding was performed at any stage of this study.

### 2.1 Preparation of Brain Slices

**FIGURE 1:**
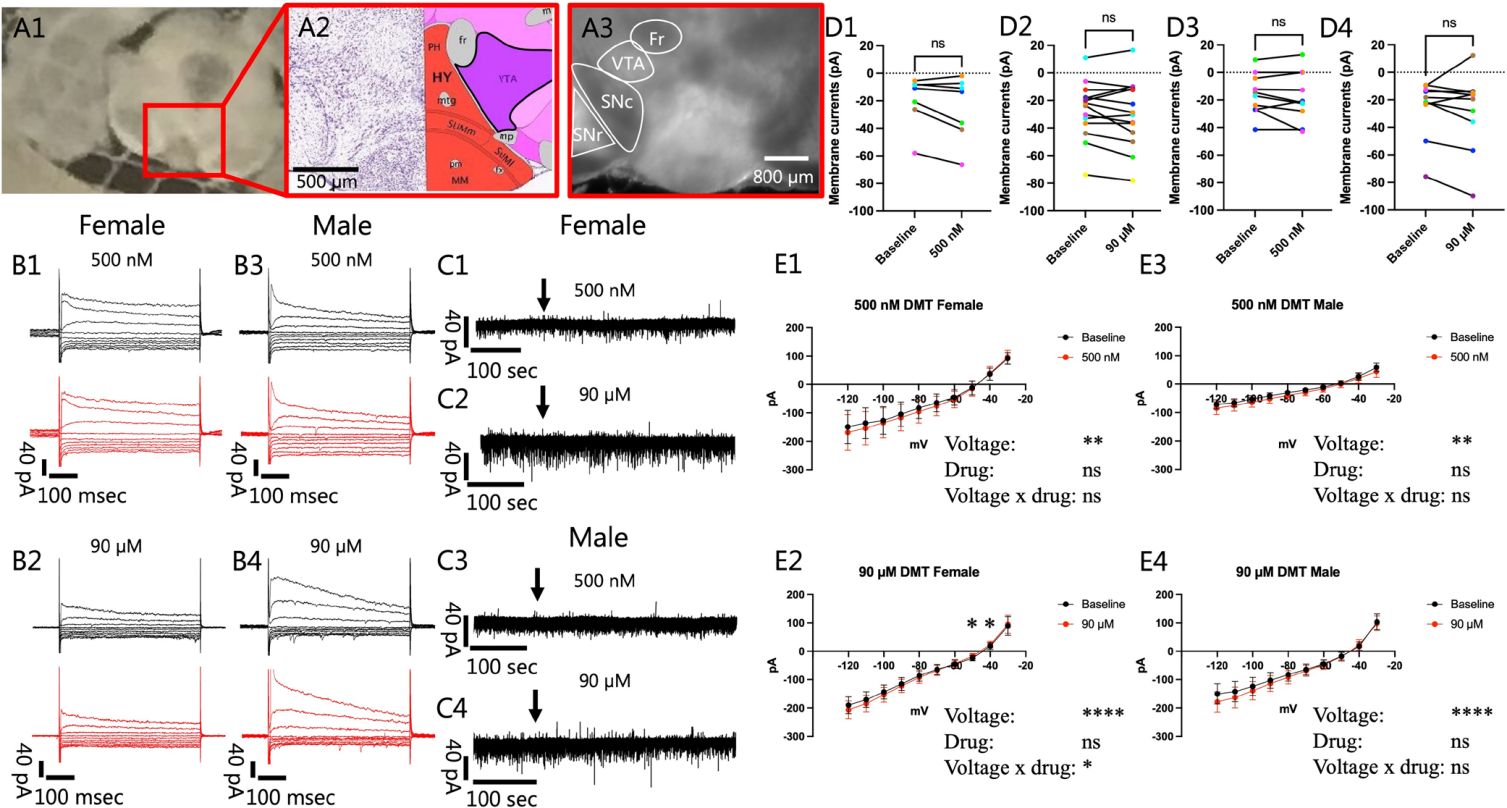
Electrophysiological recordings in voltage clamp mode from ventral tegmental area (VTA) I_h_‐negative neurons in coronal brain slices from female and male showed no effect of dimethyltryptamine (DMT) on membrane currents, but an effect on the I‐V relationship and conductance at −40 mV and −50 mV was noted in females at the higher DMT concentration. (A1) A 250 μM thick coronal brain slice is shown with the substantia nigra pars ‐compacta (SNc) and ‐reticulata (SNr) delineated with a red square, which were landmarks used for identification of the VTA. (A2) Mapping of the nuclear anatomy in a Nissl‐stained slice (Left) with corresponding anatomical notations (Right) modified from the Allen Mouse Brain Atlas, atlas.Brain‐map.org (Allen Institute for Brain Science 2004) is shown. (A3) The region of the brain boxed in A1 is shown under 4× magnification in which the SNc, SNr, Fasciculus retroflexus (Fr) and VTA are indicated. (B) Membrane current responses to voltage step increments of 10 mV showing the absence of I_h_ current under baseline conditions (Black) and during DMT (Red) application in females (1) with 500 nM and (2) with 90 μM, and (3 + 4) for males at the lower and higher DMT concentration, respectively. (C) Representative examples of a lack of change in the holding membrane currents from VTA I_h_‐negative neurons females during bath application (black arrow) of DMT at (1) 500 nM and (2) 90 μM and a lack of an effect in males (3 + 4) at the same concentrations, respectively. Analysis of the membrane currents in the population of tested cells revealed no change in membrane current following administration of both DMT concentrations in both sexes. (D) In female I_h_‐negative neurons in the VTA, no change in the holding membrane current was observed at (1) 500 nM (Baseline: −19.70 ± 6.99 pA; 500 nM: −25.30 ± 8.83 pA; *n* = 7; Two‐tailed Wilcoxon test, *W* = −18.00, *p* = 0.1562) or at (2) 90 μM (Baseline: −26.99 ± 5.51 pA; 90 μM: −29.61 ± 6.47 pA; *n* = 14; Two‐tailed paired *t*‐test, *t*(13) = 1.294, *p* = 0.2181) and the same lack of a significant change in membrane currents was observed in male neurons at (3) the lower (Baseline: −15.81 ± 4.70 pA; 500 nM: −17.76 ± 5.72 pA; *n* = 10; Two‐tailed paired *t*‐test, *t*(9) = 0.9409, *p* = 0.3713) and (4) higher (Baseline: −25.64 ± 6.68 pA; 90 μM: −27.88 ± 8.84 pA; *n* = 10; Two‐tailed Wilcoxon test, *W* = −15.00, *p* = 0.4258) DMT concentration. Same color indicates multiple cells obtained from the same brain slice. (E) Current–voltage (IV) plots of female (1 + 2) and male (3 + 4) I_h_‐negative VTA neurons in response to voltage steps from −30 mV to −120 mV before (Black) and after application (Red) of 500 nM and 90 μM are shown. As expected, voltage showed a significant main effect in both female (2‐way ANOVA, 500 nM: *F*(1.08, 5.39) = 21.6, *p* = 0.0043; Mixed‐effects analysis, 90 μM: *F*(1.35, 12.2) = 49.2, *p* < 0.0001) and male VTA I_h_‐negative neurons (2‐way ANOVA, 500 nM: *F*(1.08, 4.32) = 19.8, *p* = 0.0090; 90 μM: *F*(1.27, 10.2) = 34.1, *p* < 0.0001). At the low concentration, DMT did not exert a main effect on female VTA I_h_‐negative neurons (2‐way ANOVA, *F*(1.00, 5.00) = 0.579, *p* = 0.4809), and no interaction could be observed (2‐way ANOVA, *F*(1.37, 6.85) = 1.43, *p* = 0.2884). Increasing the concentration to 90 μM did not have a main effect (Mixed‐effects analysis, *F*(1.00, 9.00) = 0.0175, *p* = 0.8978), however, an interaction was observed, and multiple comparison testing revealed that DMT elicited a significant increase in current injected at −50 mV and at −40 mV (Mixed‐effects analysis, *F*(1.27, 9.46) = 5.55, *p* = 0.0357; Tukey's, *p*(−50 mV) = 0.0182 and *p*(−40 mV) = 0.0194). In males, no drug main effect or interaction were observed at either the low (2‐way ANOVA Drug: *F*(1.00, 4.00) = 1.49, *p* = 0.2889; interaction: *F*(1.39, 5.55) = 0.384, *p* = 0.6268) or high DMT concentration (2‐way ANOVA Drug: *F*(1.00, 8.00) = 4.36, *p* = 0.0703; interaction: *F*(1.35, 10.8) = 1.51, *p* = 0.2551). “*n*” denotes the number of neurons recorded from and error bars represents mean ± SEM. Asterisks in figures indicate differing alpha levels, *: *p* < 0.0500, **: *p* < 0.0100, ***: *p* < 0.0010, ****: *p* < 0.0001. The data obtained from cell(s) in the same brain slice are shown with the same color in D1–D4.

Eliasen et al. (2020) provides a detailed description of anesthesia, extraction of the brain, and making of brain slices using a vibratome (Eliasen et al. 2020). Mice were anesthetized in a chamber with a cotton swab soaked in 3%–5% isoflurane (Baxter; Søborg, Denmark) for 2–3 min until loss of the righting reflex and absence of response to a paw pinch. Animals were then euthanized by decapitation to confirm death before tissue collection. The brain was removed and a block of the brain containing the VTA was obtained. 250 μm coronal slices (Figure 1A1) were sectioned with a Leica VT1200S vibratome (Leica Biosystems; Wetzlar, Germany) while submerged in artificial cerebrospinal fluid (ACSF) containing (2.7 mM CaCl_2_•2H_2_O, 5 mM KCl, 26 mM NaHCO_3_, 124 mM NaCl, 1.2 mM Na_2_HPO_4_•2H_2_O, 1.2 mM MgSO_4_ (anhydrous) and 10 mM monohydrated glucose). Osmolarity and pH were adjusted, if necessary, to 300 ± 5 mOsm, 7.4 respectively, and ACSF was always saturated with carbogen. Brain slices were compared to Franklin & Paxinos (Franklin and Paxinos 2008) along with Allen's mouse brain atlas (Figure 1A2) (Allen Institute for Brain Science 2004) and were used to locate the landmarks of the substantia nigra‐ pars compacta (SNc) and pars reticulata (SNr), which ensured that VTA was present. The VTA containing brain slices were heated at 32°C with ACSF for 15 min, followed by 30–60 min of acclimatization at room temperature (~20°C–25°C), before the slices were ready for positioning under the microscope. The brain region fasciculus retroflexus (Fr) was additionally used to locate the VTA under 4× bright field optics (Figure 1A3).

### 2.2 Electrophysiology in Brain Slices

Brain slices containing VTA were placed in a RC‐26G submersion recording chamber (Warner instruments; Hamden, CT, USA) filled with and constantly perfused with oxygenated ACSF (~1.0–1.2 mL/min) using a nine‐head perfusion pump (Ole Dick; Hvidovre, Denmark), which was positioned on a stage under the objective of an upright Olympus BX51W1 microscope (Olympus Europe; Hamburg, Germany). A cooled 12‐bit PCO Sensicam CCD camera system (PCO Imaging; Kelheim, Germany) was used for visualization of the slices under bright field illumination with 4× optics (N.A. 0.1). A 40× LUMPlanFI water immersion objective (N.A. 0.8; Olympus; Tokyo, Japan) and differential‐interference‐contrast optics were used to visualize individual neurons (MacVicar 1984; Dodt et al. 2013). Neurons were selected based on soma size, with smaller round cells preferred in the VTA as potentially I_h_‐negative and presumably GABAergic neurons. Neurons were defined as lacking I_h_ currents if upon injection of current to a more negative potential from the holding potential of −60 mV (10 mV steps from −70 mV to −120 mV) at baseline failed to show hyperpolarization‐activated cyclic nucleotide–gated currents (Figure 1B). While lack of I_h_ current is commonly used for identifying GABAergic neurons in the VTA, there exists some controversy in murine (Margolis, Lock, Chefer, et al. 2006; Margolis, Lock, Hjelmstad, and Fields 2006; Margolis et al. 2012; Morales and Margolis 2017; Ford et al. 2006; Lammel et al. 2011; Ungless and Grace 2012; Zhang et al. 2010). However, in mice, the presence of I_h_ is a good proxy for dopamine neurons, as most express I_h_ current, whereas GABAergic neurons express little to no I_h_ (Lammel et al. 2008; Chieng et al. 2011).

Borosilicate glass capillaries with a filament (1.5 mm O.D. Sutter instruments; Novato, CA, USA) were pulled on a horizontal Sutter P‐97 puller (Sutter instruments; Novato, CA, USA). Pipettes were filled with a pH adjusted (7.2 pH) internal solution modified from (Hestrin and Armstrong 1996): 144 mM K‐gluconate, 3 mM MgCl_2_•6H_2_O, 10 mM HEPES, 0.3 mM Na_3_GTP, 4 mM Na_3_ATP and 200 μM Fura‐2‐penta (Invitrogen; Waltham, MA, USA). The resistance of the pipettes when filled with this solution was 7.0–10.0 MΩ.

An Axoclamp 200B amplifier (Molecular Devices; San Jose, CA, USA), which was connected to a Digi‐Data 1440A analog‐digital converter (Molecular Devices; San Jose, CA, USA) was used to obtain electrophysiological recordings under voltage‐ and current‐clamp configurations. Voltage‐ and current‐clamp data were generated using Clampex 10.2 (Molecular Devices; San Jose, CA, USA), stored and analyzed using Clampfit 10.2 (Molecular Devices; San Jose, CA, USA). Signals were collected and filtered at 20 kHz and 10 kHz, respectively, using a low‐pass Bessel filter with 70% compensation of the series resistance before each recording. The liquid junction potential was calculated to be 15 mV at room temperature, and no corrections to data were made (Neher 1992). Data from neurons were included in the analysis when the holding current at baseline was < ±100 pA, with the access resistance not exceeding 30 MΩ and with a variance not exceeding 20%. Constant single voltage square pulses of 5 mV lasting 10 msec were used to track the formation of the seal by monitoring the membrane resistance and any pipette offsets were manually corrected. Neurons that were patch clamped in the VTA were held at −60 mV once the whole‐cell configuration was obtained and a period of 5 min elapsed before data recordings began to allow for diffusion of Fura‐2‐penta into the soma and proximal dendrites.

The following protocols in voltage clamp mode were used to determine if DMT affected the membrane holding current. Firstly, the differences between holding current averages during 200 msec of baseline and an average of 200 msec sampled at least 90 s after initial DMT exposure of the slice were compared. Secondly, the current–voltage (I‐V) relationships were evaluated by stepping the holding current in incrementing steps (500 msec in duration) of +10 mV from −120 mV to −30 mV from the −60 mV holding potential. I‐V curves were obtained during baseline and ~80 s after drug entered the recording chamber. An initial fast component was observed in the majority of the recordings in both sexes and steps exhibiting this response were included in the analysis with the recordings showing no initial fast component. However, whenever breakthrough spikes were observed after the initial fast components, those steps were excluded from the analysis. Data from three female cells were partially excluded: in these cells, responses to the −30 mV and −40 mV steps were omitted. In one additional cell, only the response to the −30 mV step was excluded. In recordings without this fast component, if any action current was observed, the recording was excluded from the analysis, which resulted in one cell from the males being excluded due to this criterion. Cell conductance was derived from Ohm's law, with the conductance calculated as the DMT‐mediated change in current divided by the voltage.

Evaluation of actions of DMT on the interval and amplitudes of spontaneous excitatory postsynaptic currents (sEPSCs) was performed by comparing these two parameters between two 30 s segments, one collected immediately before DMT entered the recording chamber (Baseline) and another ~110 s after initial drug exposure. The amplitude and interval of the sEPSCs in the two (baseline and drug exposure) 30 s segments were analyzed in Minianalysis 6.0.3 (Synaptosoft Inc.; Fort Lee, NJ, USA). The threshold for detecting a synaptic event was defined as the average noise, which was measured from a section of the 30 s segments devoid of any events, multiplied by at least four times. All program detected events were manually checked.

The influence of DMT on the membrane potential, action potential firing rate, rheobase and kinetics of the afterhyperpolarization (AHP) was evaluated in current clamp mode. The membrane potential at baseline was measured from a 100 msec period just prior to drug application, which was compared to 100 msec of the membrane potential measured after ~110 s of DMT application. In a second population of cells, the membrane potential was held at just above threshold, designated as superthreshold (ST), in order to elicit reliable firing of APs, where the number, frequency, and interval of the APs were analyzed from 30 s epochs prior to DMT exposure and 30 s epochs during DMT application. Only recordings with epochs with a minimum of 3 APs during baseline and DMT application were included in the analysis; one neuron in the male was excluded due to this criterion. The interval was defined as the time between APs and the frequency was calculated as the number of APs within 30 s. In a separate population of three different groups of neurons, the membrane potential was held at ST, ST + 2–5 mV or ST + 6–9 mV where the frequency of firing of action potentials was obtained from 0.5 to 15 s segments and compared to evaluate voltage‐dependent effects of DMT on firing frequency. Effects of DMT on the rheobase, which is the minimal current injected to elicit an AP were determined by injecting incrementing 5 pA steps of positive current (duration 50 msec), with rheobase considered as the lowest current necessary to elicit a spike. The effect of DMT on the duration and amplitude of the AHP was evaluated following elicitation of single APs on a positive current step in order to avoid contamination by other APs. The amplitude of the AHP was defined as the difference in voltage from threshold to maximum polarization, whereas the duration of the AHP was defined as the time from maximum polarized potential to the threshold potential.

### 2.3 Calcium Imaging

Ratiometric fluorescence imaging was employed as an indirect method to assess changes in intracellular calcium concentrations, using both single‐cell and multi‐cell recording protocols in VTA brain slices. For single cell imaging, the ratiometric calcium indicator, Fura‐2‐penta, a cell impermeable dye was allowed to passively diffuse into the neuron through the patch pipette for 5 min before recording. For multiple cell imaging with Fura‐2‐AM, which is cell permeable, we used procedures previously reported (Eliasen et al. 2020). Briefly, VTA containing brain slices were incubated in 500 μL ACSF containing 2.5 μL of 4 mM Fura‐2‐AM (Final concentration: 20 μM; BioNordic; Herlev, Denmark) and 2.5 μL of Pluronic F‐127 (Hellobio; Dunshaughlin, Ireland) at 32°C and oxygenated (95% O_2_/5% CO_2_) for 1 min per PND. Light exposure was reduced to minimize photobleaching. The brain slice (Figure 1A1) was thereafter directly transferred to the recording chamber and continuously perfused with oxygenated ACSF to ensure de‐esterification of Fura‐2‐AM to Fura‐2 and the removal of excess dye.

By using a CCD camera and polychromator (Polychrome V; Till photonics, Germany), which were under software control (Live acquisition software 2.2.0; Till photonics, Germany), fluorescent imaging was conducted. Neurons filled with Fura‐2‐penta or Fura‐2‐AM were visualized with a Fura‐2 cube filter set (Chroma; Bellows Falls, VT, USA) when illuminated by 380 nm wavelength light. Regions of interest (ROI) were drawn to encompass somas in multiple cell imaging when using Fura‐2‐AM or around the soma of the patched neuron in single cell imaging with Fura‐2‐penta. Background was subtracted from all recordings and determined as fluorescence measured from a ROI absent of cells. Fluorescent cells were exposed to two different wavelengths during recording, 340 nm (90 msec) and 380 nm (70 msec), using a 50 msec ultrafast switch, with a frame pair interval of 4 s.

The average fluorescence (F) in each ROI was binned by 2 × 2 pixels, and following background subtraction, F at 340 nm and 380 nm was ratioed. Alterations in intracellular calcium were reflected as a shift in fluorescence. The relative change in fluorescence over time was expressed as ΔF/F_0_, where (F‐F_0_)/F_0_ = ΔF/F_0_. This value was multiplied by ×100 to express the change in percentage relative to baseline. The amplitude of the response was calculated by subtracting the average fluorescence of 10 frame pairs prior to drug application, which was defined as baseline (F_0_) from the peak response that was defined as three frame pairs showing maximum increase/decrease following DMT application. Deviations from baseline greater than 3% (ΔF/F_0_ × 100% > 3%) were considered responses and included in the analysis. All traces are presented as % ΔF/F_0_.

### 2.4 Drugs

In the majority of our studies, we used two concentrations of DMT: 500 nM and 90 μM, which was guided by pharmacologically relevant levels reported in human studies and physiological considerations from rodent experiments. Human studies suggest that endogenous DMT is present in the low nanomolar range under baseline conditions, which is well below the concentrations required to elicit overt hallucinogenic effects. When applied exogenously, such as through intravenous or vaporized routes that can induce profound psychedelic experiences associated with therapeutic actions, brain concentrations are estimated to transiently reach low micromolar levels. We wished to use concentrations sufficient to achieve clinically relevant neural exposures. However, complicating this is a difference in plasma and brain levels between humans and rodents. When DMT plasma levels in humans typically reach ∼0.06–0.5 μM following an antidepressant dose, rodent brain tissue concentrations can be much higher (~90 μM) following an antidepressant dose (Ly et al. 2018; Carbonaro and Gatch 2016; Cohen and Vogel 1972). Additionally, *i.p*. administration of 5 mg/kg DMT in rats resulted in a 6 times greater concentration in brain when compared to that in plasma (Cohen and Vogel 1972). This suggests that local brain concentrations can be significantly higher than plasma levels and therefore, when using rodent tissue, high concentrations should also be evaluated. Additionally, increased frequency and amplitude of sEPSCs observed 24 h in the PFC after DMT administration at both hallucinogenic (10 mg/kg) and sub‐hallucinogenic (1 mg/kg) doses in rats indicate lasting neurophysiological alterations beyond the compound's metabolic presence (Ly et al. 2018). When these hallucinogenic and sub‐hallucinogenic doses are administered (*i.p*. and *s.c*., respectively), they correspond roughly to 45–51 μM (Cohen and Vogel 1972; Shah and Hedden 1978) and 0.55 (cerebellum)–0.8 μM (frontal cortex) (Egger et al. 2023), respectively, when assuming brain density of 1 g/mL. In order to be guided by a physiological basis, we decided to use 0.5 μM (500 nM) to mimic the upper antidepressant plasma concentrations observed in humans given a psychedelic dose and the antidepressant action measured in murine brain tissues (90 μM) (Cohen and Vogel 1972; Cameron et al. 2018; Callaway et al. 1999; Riba et al. 2003; Yritia et al. 2002), while also being in the reported sub‐hallucinogenic and hallucinogenic region, respectively. The lower concentration corresponds to the upper affinity of the 5‐HT_2A_ receptor (~462 nM) (Sadzot et al. 1989), whereas the higher concentration likely involves additional receptors. In follow‐up work, we evaluated an intermediate concentration (10 μM) in order to determine dose‐dependent actions of DMT on firing.

Accordingly, DMT (Cayman chemical; Ann Arbor, MI, USA) was dissolved in 96% ethanol to a final stock solution of 500 μM, 10 μM, or 90 mM. On the experiment day, the stock solution was diluted ×1000 in ACSF to a final concentration of 500 nM, 10 μM, or 90 μM, which resulted in a final concentration of ethanol that we have shown in preliminary studies does not alter cellular activity. In both voltage‐ and current‐clamp mode, baseline was monitored for a minimum of 60 s before bath applying DMT (5 mL). DMT was applied with a duration of 250–300 s. As DMT is an amine, it is rapidly degraded by monoamine oxidase (MAO) in the gastrointestinal tract, which necessitates concurrent use of MAO inhibitors for effect when ingested orally. However, we did not use MAO inhibitors as we did not expect sufficient enzymatic degradation as supported by studies showing that when smoked, DMT circumvents degradation in the gut and intestine by directly entering the bloodstream, where it readily acts on the brain (Barker 2022; Riba et al. 2015).

### 2.5 Statistics

All statistical analyses were carried out in GraphPad Prism version 10 (San Diego, CA, USA). Data were tested for normality using the Shapiro–Wilk test for normality. Depending on the outcome of normality testing, a two‐tailed paired *t*‐test or Wilcoxon test was employed. I‐V relationship and conductance changes were investigated using a 2‐way ANOVA with repeated measures or when data points were removed due to contamination by fast‐inactivating or active currents, mixed‐effects analysis was utilized and, when indicated, post hoc testing was conducted with Tukey's multiple comparison. Statistical analysis of the amplitudes of changes of ΔF/F_0_ was conducted on changes in fluorescence normalized to baseline (100%) values. *p*‐values < 0.0500 were considered significant and asterisks in the figures display differing alpha levels; *: *p* < 0.0500, **: *p* < 0.0100, ***: *p* < 0.0010, ****: *p* < 0.0001. No test for outliers was conducted. A power analysis was not conducted in order to predetermine the sample size prior to experimentation. The number of cells included in this study was determined based on prior experiments conducted in our laboratory using similar experimental designs and outcome measures. Under identical conditions and evaluating identical parameters, a previous study from our laboratory revealed differences with a similar sample size, and in another study from our laboratory, data sets with a similar sample size detected robust effects (Eliasen et al. 2025a; Yang et al. 2025). All results are presented as mean ± SEM and ‘*n*’ denotes the number of individual neurons from which data sourced. Most data sets contained data obtained from at least three mice of each sex; however, for the multiple cell imaging and firing rate conducted with 10 μM, only two mice per sex were used. All analyses assessing the effects of DMT were performed on data collected during drug application. Figures were prepared using Igor Pro software 6.20 (Wavemetrics; Portland, OR, USA) and Graphpad Prism 10.0.

## 3 Results

VTA brain slices from 37 female and 29 male NMRI mice were used for this study. Anatomical landmarks in the coronal brain slices were identified (Figure 1A1–A3) to ensure the recordings derived from VTA neurons. Data sourced from neurons which failed to exhibit I_h_ currents upon injection of current to a more negative potential from the holding potential of −60 mV (10 mV steps from −70 mV to −120 mV) at baseline and during DMT application of two concentrations of DMT (Figure 1B1–B4).

### 3.1 Membrane Current and Conductance Were Not Altered by Two DMT Concentrations in Female and Male I_h_‐Negative VTA Neurons

The electrophysiological responses of I_h_‐negative VTA neurons to bath application of 500 nM and 90 μM of DMT were monitored in voltage‐clamp mode (Figure 1C1–C4). Both concentrations of DMT failed to induce a change in membrane current in both sexes under our recording conditions. In female neurons, there was no statistical difference between the average baseline holding current and the holding current recorded during application of 500 nM DMT (Baseline: −19.70 ± 6.99 pA; 500 nM: −25.30 ± 8.83 pA; *n* = 7; Two‐tailed Wilcoxon test, *W* = −18.00, *p* = 0.1562; Figure 1D1) or during application of 90 μM DMT (Baseline: −26.99 ± 5.51 pA; 90 μM: −29.61 ± 6.47 pA; *n* = 14; Two‐tailed paired *t*‐test, *t*(13) = 1.294, *p* = 0.2181; Figure 1D2). Similarly, the average membrane holding current did not significantly change in male VTA neurons when exposed to the lower DMT concentration (Baseline: −15.81 ± 4.70 pA; 500 nM: −17.76 ± 5.72 pA; *n* = 10; Two‐tailed paired *t*‐test, *t*(9) = 0.9409, *p* = 0.3713; Figure 1D3) or to the higher DMT concentration (Baseline: −25.64 ± 6.68 pA; 90 μM: −27.88 ± 8.84 pA; *n* = 10; Two‐tailed Wilcoxon test, *W* = −15.00, *p* = 0.4258; Figure 1D4).

Although we did not observe an effect on holding membrane current, we evaluated the I‐V relationship and potential conductance changes to determine whether voltage‐dependent actions were elicited. In the I‐V relationship, and as expected, the voltage was a significant main effect at both concentrations (2‐way ANOVA, 500 nM: *F*(1.08, 5.39) = 21.6, *p* = 0.0043; Mixed‐effects analysis, 90 μM: *F*(1.35, 12.2) = 49.2, *p* < 0.0001). However, the application of DMT to female VTA neurons at both 500 nM (2‐way ANOVA, *F*(1.00, 5.00) = 0.579, *p* = 0.4809; Figure 1E1) and 90 μM (Mixed‐effects analysis, *F*(1.00, 9.00) = 0.0175, *p* = 0.8978; Figure 1E2) was not associated with any significant main effect. In females, applying 500 nM of DMT did not result in a significant interaction between voltage × DMT (2‐way ANOVA, *F*(1.37, 6.85) = 1.43, *p* = 0.2884), but at the higher concentration, an interaction effect was apparent, which was driven by a significant increased current necessary to hold the cell at the subthreshold potentials of −40 and −50 mV with 90 μM of DMT (Mixed‐effects analysis, *F*(1.27, 9.46) = 5.55, *p* = 0.0357; Tukey's, *p*(−50 mV) = 0.0182 and *p*(−40 mV) = 0.0194). In males, voltage exerted a significant main effect both when applying 500 nM (2‐way ANOVA, *F*(1.08, 4.32) = 19.8, *p* = 0.0090; Figure 1E3) and 90 μM of DMT (2‐way ANOVA, *F*(1.27, 10.2) = 34.1, *p* < 0.0001; Figure 1E4). However, at both 500 nM or 90 μM, DMT did not have a significant effect (2‐way ANOVA, 500 nM: *F*(1.00, 4.00) = 1.49, *p* = 0.2889; 2‐way ANOVA, 90 μM: *F*(1.00, 8.00) = 4.36, *p* = 0.0703), and no interactions were detected (2‐way ANOVA, 500 nM: *F*(1.39, 5.55) = 0.384, *p* = 0.6268; 2‐way ANOVA, 90 μM: *F*(1.35, 10.8) = 1.51, *p* = 0.2551).

To provide complementary insight, we evaluated the conductance at each voltage step. Voltage was a significant main effect at both concentrations in both female (2‐way ANOVA, 500 nM: *F*(1.07, 5.34) = 46.8, *p* = 0.0007; Mixed‐effects analysis, 90 μM: *F*(1.13, 10.2) = 33.5, *p* = 0.0001) and male (2‐way ANOVA, 500 nM: *F*(1.09, 4.36) = 14.4, *p* = 0.0157; 2‐way ANOVA, 90 μM: *F*(1.14, 9.12) = 28.2, *p* = 0.0004) mice. Voltage was the only parameter that was significant in males, as DMT at either 500 nM or 90 μM showed a significant main effect (2‐way ANOVA, 500 nM: *F*(1.00, 4.36) = 14.4, *p* = 0.2840; 2‐way ANOVA, 90 μM: *F*(1.00, 8.00) = 4.21, *p* = 0.0742) or interaction between DMT × voltage (2‐way ANOVA, 500 nM: *F*(1.04, 4.14) = 0.690, *p* = 0.4565; 2‐way ANOVA, 90 μM: *F*(1.11, 8.91) = 0.283, *p* = 0.6321). In females, DMT at both the low and high concentrations failed to exert a significant effect on the conductance (2‐way ANOVA, 500 nM: *F*(1.00, 5.00) = 0.333, *p* = 0.5891; Mixed‐effects analysis, 90 μM: *F*(1.00, 9.00) = 8.42 × 10^−7^, *p* = 0.9993). At the low concentration, no interaction was present in female neurons (2‐way ANOVA, *F*(1.51, 7.54) = 2.39, *p* = 0.1610). However, 90 μM of DMT showed a significant interaction, driven by an increase in conductance during DMT application at −50 mV and −40 mV (Mixed‐effects analysis, *F*(1.66, 12.4) = 7.09, *p* = 0.0113; Tukey's, *p*(−50 mV) = 0.0182 and *p*(−40 mV) = 0.0193), which collaborated findings seen in evaluation of the I‐V relationship. Taken together, 90 μM DMT produced a significant effect on the I‐V curve of females at −40 and −50 mV, which was accompanied by a corresponding significant change in conductance, indicating that DMT alters both current amplitude and voltage‐dependent channel properties in a sex and concentration‐based fashion.

### 3.2 500 nM and 90 μM DMT Failed to Alter EPSCs Interval or Amplitude in Either Sex

**FIGURE 2:**
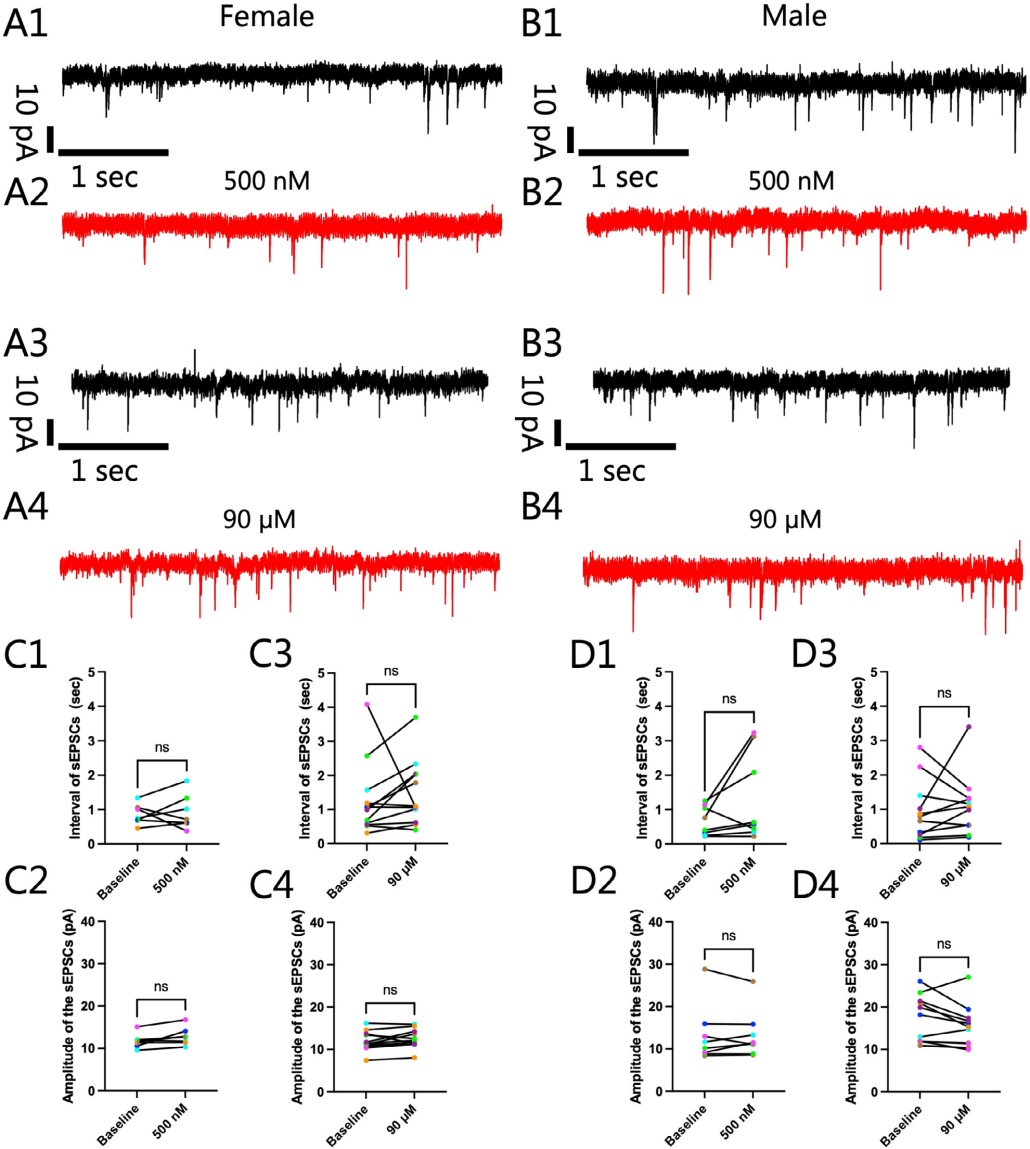
Applying either 500 nM or 90 μM of dimethyltryptamine (DMT) to female and male I_h_‐negative ventral tegmental area (VTA) neurons did not change the interval or amplitude of spontaneous excitatory postsynaptic currents (sEPSCs). Representative voltage clamp mode recordings of sEPSCs at baseline and following 500 nM and 90 μM of DMT for females (A1–A4) and males (B1–B4), respectively. In the female, VTA I_h_‐negative neurons did not show a significant change in the average (C1) interval (Baseline: 0.86 ± 0.11 s; 500 nM: 0.93 ± 0.19 s; *n* = 7; Two‐tailed paired *t*‐test, *t*(6) = 0.4141, *p* = 0.6932) or (C2) amplitude (Baseline: 11.72 ± 0.65 pA; 500 nM: 12.84 ± 0.79 pA; *n* = 7; Two‐tailed paired *t*‐test, *t*(6) = 2.398, *p* = 0.0534) of sEPSCs when 500 nM DMT was applied. Increasing the DMT concentration to 90 μM also failed to induce a change in female I_h_‐negative neurons (C3) EPSCs intervals (Baseline: 1.27 ± 0.31 s; 90 μM: 1.48 ± 0.27 s; *n* = 12; Two‐tailed Wilcoxon test, *W* = 38.00, *p* = 0.1514) or sEPSC (C4) amplitudes (Baseline: 11.91 ± 0.65 pA; 90 μM: 12.38 ± 0.63 pA; *n* = 12; Two‐tailed paired *t*‐test, *t*(11) = 1.226, *p* = 0.2459). Similarly, when 500 nM DMT was applied to male I_h_‐negative neurons, a change was not seen in the average sEPSC (D1) interval (Baseline: 0.68 ± 0.15 s; 500 nM: 1.33 ± 0.45 s; *n* = 8; Two‐tailed Wilcoxon test, *W* = 24.00, *p* = 0.1094) or (D2) amplitude (Baseline: 13.24 ± 2.40 pA; 500 nM: 13.26 ± 1.98 pA; *n* = 8; Two‐tailed Wilcoxon test, *W* = 2.00, *p* = 0.9453). Increasing the concentration also failed to elicit a significant change in the (D3) interval (Baseline: 0.97 ± 0.26 s; 90 μM: 1.12 ± 0.27 s; *n* = 11; Two‐tailed Wilcoxon test, *W* = 10.00, *p* = 0.7002) or (D4) amplitude (Baseline: 17.20 ± 1.65 pA; 90 μM: 15.43 ± 1.49 pA; *n* = 11; Two‐tailed Paired *t*‐test, *t*(10) = 1.939, *p* = 0.0813) of the sEPSCs. “*n*” denotes the number of neurons recorded from and error bars represents mean ± SEM. The data obtained from cell(s) in the same brain slice are shown with the same color in C1–C4 and D1–D4.

We next evaluated whether DMT influenced excitatory synaptic events (Figure 2; Female A1–A4; Male B1–B4). In females at the low DMT concentration, we did not detect a significant difference in the interval (Baseline: 0.86 ± 0.11 s; 500 nM: 0.93 ± 0.19 s; *n* = 7; Two‐tailed paired *t*‐test, *t*(6) = 0.4141, *p* = 0.6932; Figure 2C1), or the amplitude of sEPSCs (Baseline: 11.72 ± 0.65 pA; 500 nM: 12.84 ± 0.79 pA; *n* = 7; Two‐tailed paired *t*‐test, *t*(6) = 2.398, *p* = 0.0534; Figure 2C2). At the higher concentration, DMT also failed to induce any significant change in the amplitude (Baseline: 11.91 ± 0.65 pA; 90 μM: 12.38 ± 0.63 pA; *n* = 12; Two‐tailed paired *t*‐test, *t*(11) = 1.226, *p* = 0.2459; Figure 2C4) or interval (Baseline: 1.27 ± 0.31 s; 90 μM: 1.48 ± 0.27 s; *n* = 12; Two‐tailed Wilcoxon test, *W* = 38.00, *p* = 0.1514; Figure 2C3) of sEPSCs. Similarly, in male VTA, the intervals of sEPSCs were not altered by the lower DMT concentration (Baseline: 0.68 ± 0.15 s; 500 nM: 1.33 ± 0.45 s; *n* = 8; Two‐tailed Wilcoxon test, *W* = 24.00, *p* = 0.1094; Figure 2D1) and there was no difference in the average sEPSC amplitude (Baseline: 13.24 ± 2.40 pA; 500 nM: 13.26 ± 1.98 pA; *n* = 8; Two‐tailed Wilcoxon test, *W* = 2.00, *p* = 0.9453; Figure 2D2) of the sEPSCs. At the higher concentration, there were no significant differences observed in the sEPSC interval (Baseline: 0.97 ± 0.26 s; 90 μM: 1.12 ± 0.28 s; *n* = 11; Two‐tailed Wilcoxon test, *W* = 10.00, *p* = 0.7002; Figure 2D3) or sEPSC amplitude (Baseline: 17.20 ± 1.65 pA; 90 μM: 15.43 ± 1.49 pA; *n* = 11; Two‐tailed paired *t*‐test, *t*(10) = 1.939, *p* = 0.0813; Figure 2D4) in males. Taken together, our data suggest that acute application of DMT at the concentrations evaluated does not alter excitatory transmission directed to female or male I_h_‐negative VTA neurons.

### 3.3 90 μM DMT Did Not Affect the Membrane Potential in Both Sexes but Was Associated With an Increase in the Firing Rate in Females

**FIGURE 3:**
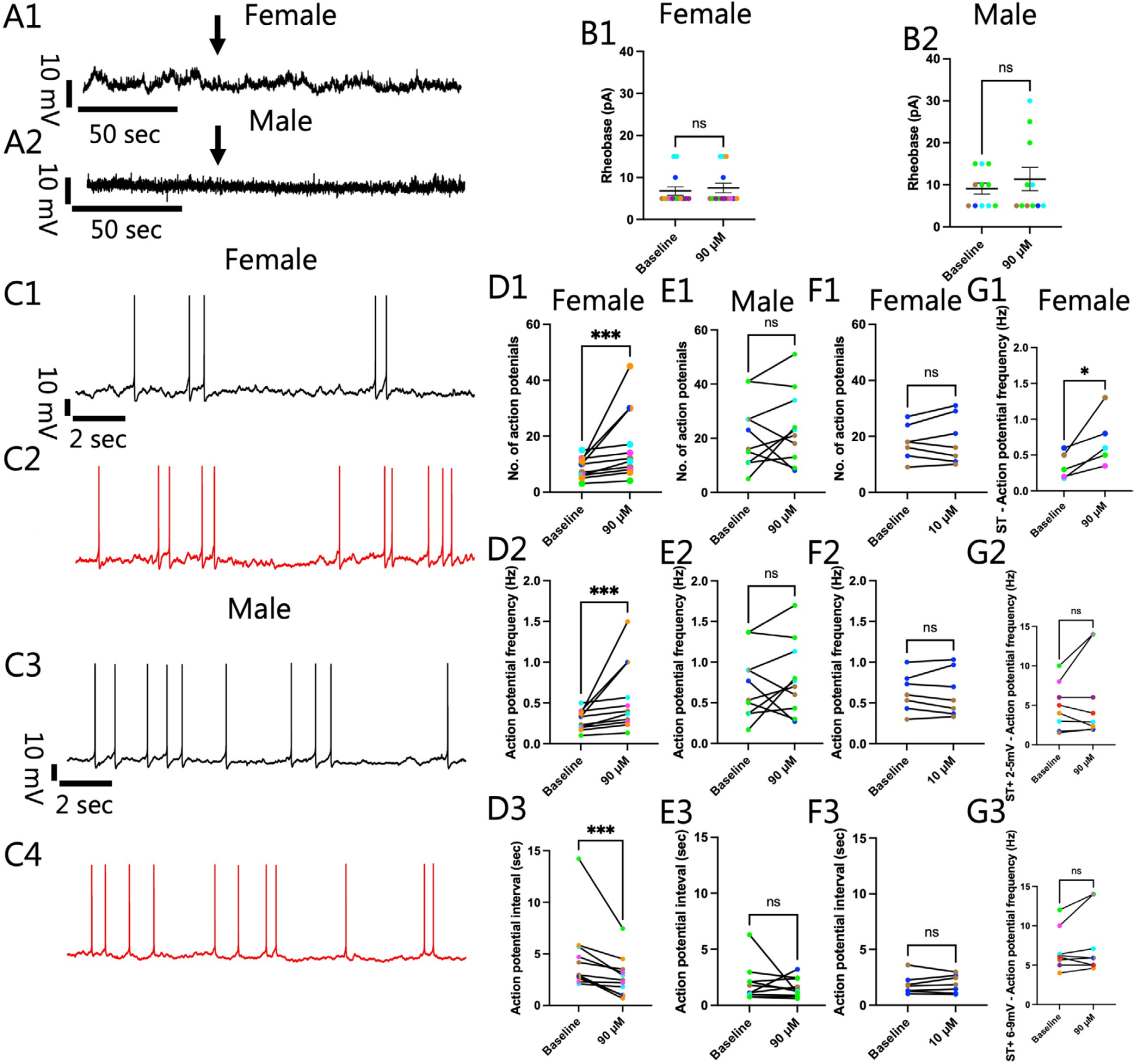
90 μM Dimethyltryptamine (DMT) failed to induce a change in membrane potential in ventral tegmental area (VTA) I_h_‐negative neurons in current‐clamp recordings in males or females; however, a sex‐based difference was noted as a significant change in the number, frequency and interval of action potentials (APs) was elicited in females that was not seen in males. (A) Current clamp recordings from representative I_h_‐negative VTA neurons cells reflecting a lack of change in membrane current seen upon DMT exposure that was also reflected in the population of cells from (1) female (Baseline: −54.4 ± 8.2 mV; 90 μM: −54.0 ± 8.6 mV; *n* = 3; Two‐tailed paired *t*‐test, *t*(2) = 0.6997, *p* = 0.5565) and (2) male (Baseline: −53.8 ± 4.7 mV; 90 μM: −54.51 ± 5.6 mV; *n* = 3; Two‐tailed paired *t*‐test, *t*(2) = 2.678, *p* = 0.1158). Arrows in representative recordings indicate the onset of DMT exposure. (B) Differences were not detected in rheobase between baseline and during DMT application for (1) female (Baseline: 6.79 ± 1.00 pA; 90 μM: 7.50 ± 1.14; *n* = 14; Two‐tailed Wilcoxon test, *W* = 1.00, *p* > 0.9999) or male (Baseline: 9.09 ± 1.32 pA; 90 μM: 11.36 ± 2.79; *n* = 11; Two‐tailed Wilcoxon test, *W* = 10.00, *p* = 0.4062) neurons. (C) Representative recordings of AP firing at baseline (Female: C1; Male: C3) and during DMT application (Female: C2; Male: C4) are shown in black and red, respectively. (D) Analyses of the number of APs showed that (D1) 90 μM of DMT induced a significant increase in the number of APs in female VTA I_h_‐negative neurons (Baseline: 8.82 ± 1.10 APs; 90 μM: 17.00 ± 3.82 APs; *n* = 11; Two‐tailed Wilcoxon test, *W* = 66.00, *p* = 0.0010). (D2) This was also reflected in an increase in the frequency of AP firing (Baseline: 0.29 ± 0.04 Hz; 90 μM: 0.57 ± 0.13 Hz; *n* = 11; Two‐tailed Wilcoxon test, *W* = 66.00, *p* = 0.0010). In addition, and as expected, (D3) the interval was significantly lower during DMT application (Baseline: 4.57 ± 1.04 s; 90 μM: 2.79 ± 0.59 s; *n* = 11; Two‐tailed Wilcoxon test, *W* = −66.00, *p* = 0.0010). In contrast, when evoking APs in male I_h_‐negative VTA neurons, there were no significant differences in (E1) the number (Baseline: 21.70 ± 3.93 APs; 90 μM: 24.00 ± 4.35 APs; *n* = 10; Two‐tailed paired *t*‐test, *t*(9) = 0.6984, *p* = 0.5026), (E2) the frequency (Baseline: 0.72 ± 0.13 Hz; 90 μM: 0.80 ± 0.14 Hz; *n* = 10; Two‐tailed paired *t*‐test, *t*(9) = 0.6970, *p* = 0.5034) or (E3) the interval (Baseline: 1.98 ± 0.53 s; 90 μM: 1.57 ± 0.27 s; *n* = 10; Two‐tailed Wilcoxon test, *W* = −12.00, *p* = 0.5742) of APs. Comparing the firing rate between baseline and during 10 μM of DMT, there was no difference in (F1) the number (Baseline: 17.86 ± 2.32 APs; 10 μM: 18.71 ± 3.23 APs; *n* = 7; Two‐tailed paired *t*‐test, *t*(6) = 0.7006, *p* = 0.5098), (F2) frequency (Baseline: 0.63 ± 0.09 Hz; 10 μM: 0.62 ± 0.11 Hz; *n* = 7; Two‐tailed paired *t*‐test, *t*(6) = 0.1382, *p* = 0.8946) or (F3) intervals (Baseline: 1.86 ± 0.33 s; 10 μM: 1.92 ± 0.30 s; *n* = 7; Two‐tailed paired *t*‐test, *t*(6) = 0.4162, *p* = 0.6917) of the APs. In a separate population of neurons from those presented in D, when held at suprathreshold (ST), (G1) the frequency of firing of APs (Baseline: 0.36 ± 0.08 Hz; 90 μM: 0.71 ± 0.16 Hz; *n* = 5; Two‐tailed paired *t*‐test, *t*(4) = 2.928, *p* = 0.0429) was significantly higher during DMT application, which was consistent with the earlier data set. The effect of DMT on the firing rate could not be observed when holding another populations of female VTA I_h_‐negative neurons cells at a more depolarized potential, namely (G2) ST + 2–5 mV (Baseline: 4.90 ± 1.06 Hz; 90 μM: 5.89 ± 1.83 Hz; *n* = 8; Two‐tailed Wilcoxon test, *W* = 8.00, *p* = 0.5781) or in a third group of female VTA I_h_‐negative neurons cells held at (G3) ST + 6–9 mV (Baseline: 6.91 ± 0.95 Hz; 90 μM: 7.70 ± 1.40 Hz; *n* = 8; Two‐tailed Wilcoxon test, *W* = 14.00, *p* = 0.2969). “*n*” denotes the number of neurons recorded from and error bars represents mean ± SEM. Asterisks in figures indicate differing alpha levels, *: *p* < 0.0500, **: *p* < 0.0100, ***: *p* < 0.0010, ****: *p* < 0.0001. B1, D1–D3 are from female and B2, E1–E3 are from males. The data obtained from cell(s) in the same brain slice are shown with the same color in B1, B2, D1–D3, and E1–E3.

Our next step was to evaluate whether electrophysiological actions could be seen in current clamp mode. As we had seen no differences at the lower concentrations of DMT in our voltage clamp experiments, we tested only the higher concentration in this series of studies. Accordingly, in current clamp mode, we evaluated whether alterations in the membrane potential of female and male VTA I_h_‐negative neurons were evoked when cells were held at their resting membrane potential i.e., when holding currents = 0, when 90 μM DMT was applied (Figure 3A1,A2). Consistent with results seen in voltage clamp mode, 90 μM DMT did not significantly alter the average membrane potential in either female or male VTA neurons. The average membrane potential in female VTA neurons was −54.4 ± 8.2 mV and −54.0 ± 8.6 mV (*n* = 3) at baseline and during DMT application, respectively, which did not represent a significant difference (Two‐tailed paired *t*‐test, *t*(2) = 0.6997, *p* = 0.5565; Figure 3A1). The average membrane potential of male VTA neurons during application of 90 μM DMT was −54.5 ± 5.6 mV (*n* = 3), which was not significantly different from the average potential measured during baseline (Baseline: −53.8 ± 4.7 mV; *n* = 3; Two‐tailed paired *t*‐test, *t*(2) = 2.678, *p* = 0.1158; Figure 3A2).

While there was no effect of DMT on membrane potential, to determine if exposure of DMT resulted in a change in excitability in VTA I_h_‐negative cells, we evaluated rheobase, a measure of the minimum current required to elicit an action potential (Figure 3B1,B2). DMT did not result in any significant change in the average rheobase in females (Baseline: 6.79 ± 1.00 pA; 90 μM: 7.50 ± 1.14 pA; *n* = 14; Two‐tailed Wilcoxon test, *W* = 1.00, *p* > 0.9999; Figure 3B1) or males (Baseline: 9.09 ± 1.32 pA; 90 μM: 11.36 ± 2.79 pA; *n* = 11; Two‐tailed Wilcoxon test, *W* = 10.00, *p* = 0.4062; Figure 2B2).

To investigate whether DMT affected AP firing, neurons were held at a depolarized potential just sufficient to elicit multiple APs and parameters of firing were evaluated (Figure 3C1–C4). At the ST potential for eliciting APs in females (Female: −40.98 ± 1.51 mV; *n* = 11), we observed that DMT significantly increased the number (Baseline: 8.82 ± 1.10 APs; 90 μM: 17.00 ± 3.82 APs; *n* = 11; Two‐tailed Wilcoxon test, *W* = 66.00, *p* = 0.0010; Figure 3D1) and frequency (Baseline: 0.29 ± 0.04 Hz; 90 μM: 0.57 ± 0.13 Hz; *n* = 11; Two‐tailed Wilcoxon test, *W* = 66.00, *p* = 0.0010; Figure 3D2) of APs, which corresponded to a significant decrease in AP intervals (Baseline: 4.57 ± 1.04 s; 90 μM: 2.79 ± 0.59 s; *n* = 11; Two‐tailed Wilcoxon test, *W* = −66.00, *p* = 0.0010; Figure 3D3). However, when male neurons were held at ST for evoking APs (Male: −34.51 ± 4.93 mV; *n* = 10), there were no significant differences observed in the number (Baseline: 21.70 ± 3.93 APs; 90 μM: 24.00 ± 4.35 APs; *n* = 10; Two‐tailed paired *t*‐test, *t*(9) = 0.6984, *p* = 0.5026; Figure 3E1), frequency (Baseline: 0.72 ± 0.13 Hz; 90 μM: 0.80 ± 0.14 Hz; *n* = 10; Two‐tailed paired *t*‐test, *t*(9) = 0.6970, *p* = 0.5034; Figure 3E2) or intervals (Baseline: 1.98 ± 0.53 s; 90 μM: 1.57 ± 0.27 s; *n* = 10; Two‐tailed Wilcoxon test, *W* = −12.00, *p* = 0.5742; Figure 3E3) of APs during application of DMT when compared to baseline conditions.

To assess dose and voltage dependence of the effect of DMT on firing, we performed additional experiments in separate groups of cells. Dose dependence was examined in one group using a~10‐fold lower concentration. There was no difference in the number (Baseline: 17.86 ± 2.32 APs; 10 μM: 18.71 ± 3.23 APs; *n* = 7; Two‐tailed paired *t*‐test, *t*(6) = 0.7006, *p* = 0.5098; Figure 3F1), frequency (Baseline: 0.63 ± 0.09 Hz; 10 μM: 0.62 ± 0.11 Hz; *n* = 7; Two‐tailed paired *t*‐test, *t*(6) = 0.1382, *p* = 0.8946; Figure 3F2) or intervals (Baseline: 1.86 ± 0.33 s; 10 μM: 1.92 ± 0.30 s; *n* = 7; Two‐tailed paired *t*‐test, *t*(6) = 0.4162, *p* = 0.6917; Figure 3F3) of APs between baseline conditions and 10 μM application of DMT, indicating that the changes in firing occur within the 10–90 μM range. Voltage dependence was tested in three separate groups of cells, with each group tested over distinct voltage strengths by applying three current strengths in order to elicit firing: ST, ST +2–5 mV, and ST + 6–9 mV to evoke action potentials. In female VTA I_h_‐negative neurons held at ST, AP frequency (Baseline: 0.36 ± 0.08 Hz; 90 μM: 0.71 ± 0.16 Hz; *n* = 5; Two‐tailed paired *t*‐test, *t*(4) = 2.928, *p* = 0.0429; Figure 3G1) was significantly higher during DMT application, replicating the effect observed in the earlier data set. An effect of DMT on the firing rate in female VTA I_h_‐negative neurons could not be observed when holding two different groups of cells at more depolarized potentials, namely ST + 2–5 mV (Baseline: 4.90 ± 1.06 Hz; 90 μM: 5.89 ± 1.83 Hz; *n* = 8; Two‐tailed Wilcoxon test, *W* = 8.00, *p* = 0.5781; Figure 3G2) or ST + 6–9 mV (Baseline: 6.91 ± 0.95 Hz; 90 μM: 7.70 ± 1.40 Hz; *n* = 8; Two‐tailed Wilcoxon test, *W* = 14.00, *p* = 0.2969; Figure 3G3).

**FIGURE 4:**
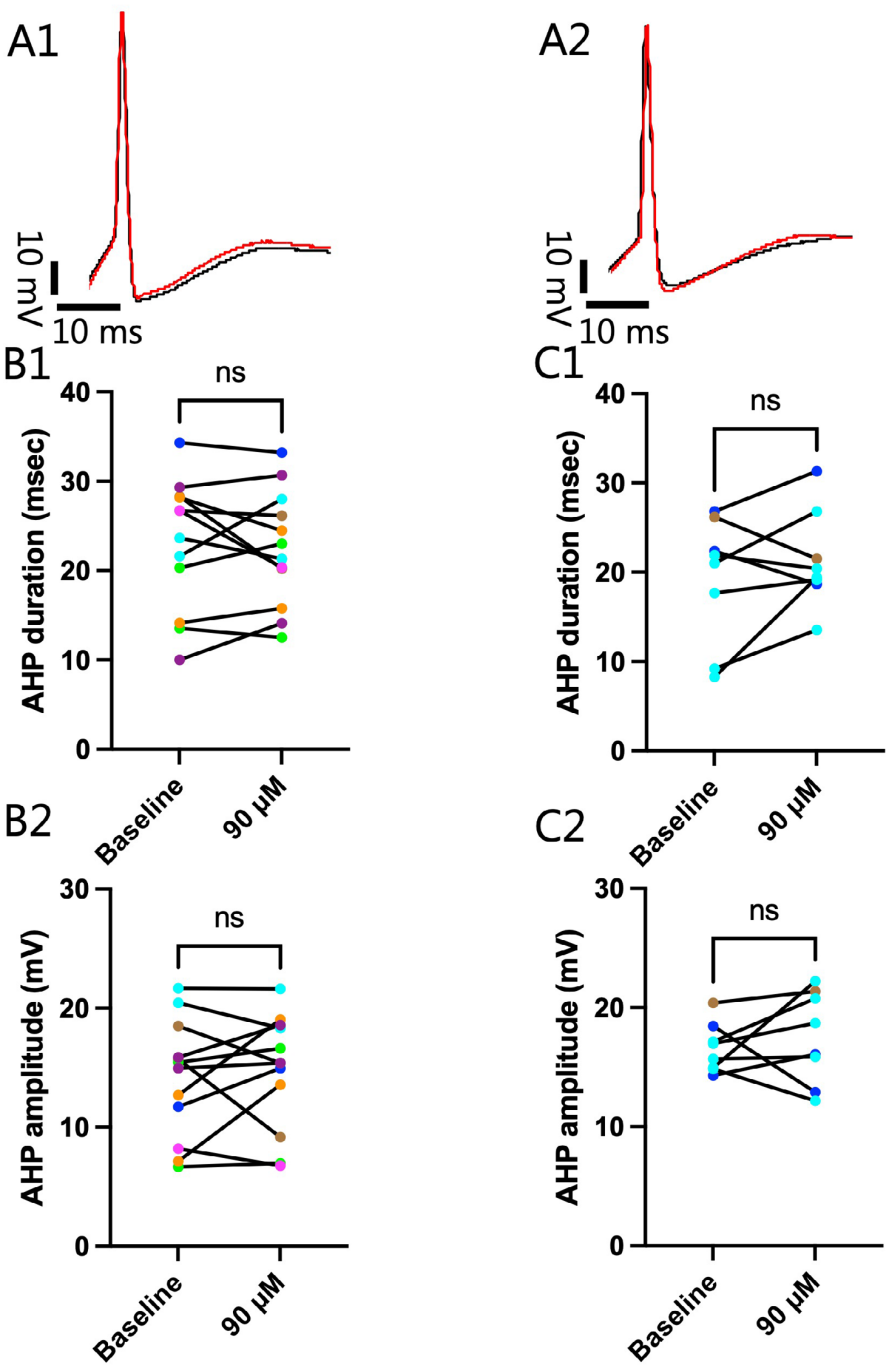
No differences in afterhyperpolarization (AHP) kinetics in either female or male ventral tegmental area (VTA) I_h_‐negative neurons were induced by exposure to 90 μM dimethyltryptamine (DMT). (A) Representative action potentials (APs) in (1) females and (2) males at baseline (Black) and during 90 μM DMT application (Red) are overlaid showing that AHP kinetics appeared very similar before and after drug. (B1) Following DMT exposure in female VTA I_h_‐negative neurons, there was no difference in the average AHP duration (Baseline: 23.07 ± 2.12 msec; 90 μM: 22.49 ± 1.86 msec; *n* = 12; Two‐tailed paired *t*‐test, *t*(11) = 0.4814, *p* = 0.6396) or (B2) amplitude (Baseline: 14.08 ± 1.44 mV; 90 μM: 14.69 ± 1.39 mV; *n* = 12; Two‐tailed paired *t*‐test, *t*(11) = 0.5622, *p* = 0.5852). Likewise, (C2) 90 μM of DMT did not induce a change in the average AHP duration (Baseline: 19.19 ± 2.50 msec; 90 μM: 21.36 ± 1.92 msec; *n* = 8; Two‐tailed paired *t*‐test, *t*(7) = 1.150, *p* = 0.2880) or (C2) amplitude (Baseline: 16.59 ± 0.73 mV; 90 μM: 17.50 ± 1.36 mV; *n* = 8; Two‐tailed paired *t*‐test, *t*(7) = 0.6661, *p* = 0.5267) in male I_h_‐negative VTA neurons. “*n*” denotes the number of neurons recorded from and error bars represents mean ± SEM. The data obtained from cell(s) in the same brain slice are shown with the same color in B1, B2 and C1, C2.

To explore one possible underlying mechanism for the DMT induced alteration in the firing rate seen in females, we evaluated the effects of 90 μM DMT on the AHP duration and amplitude of APs elicited at ST (Figure 4A1,A2), as these two kinetics can influence the timing of APs. Contrary to what was expected, no influence of DMT could be observed in female VTA neurons in either the average AHP duration (Baseline: 23.07 ± 2.12 msec; 90 μM: 22.49 ± 1.86 msec; *n* = 12; Two‐tailed paired *t*‐test, *t*(11) = 0.4814, *p* = 0.6396; Figure 4B1) or average AHP amplitude (Baseline: 14.08 ± 1.44 mV; 90 μM: 14.69 ± 1.39 mV; *n* = 12; Two‐tailed paired *t*‐test, *t*(11) = 0.5622, *p* = 0.5852; Figure 4B2). These results suggest that alterations in the kinetics of the AHP are not responsible for the alteration in firing rate seen in females following DMT exposure. We also evaluated the AHP kinetics in male VTA neurons and found that the average AHP duration (Baseline: 19.19 ± 2.50 msec; 90 μM: 21.36 ± 1.92 msec; *n* = 8; Two‐tailed paired *t*‐test, *t*(7) = 1.150, *p* = 0.2880; Figure 4C1) and average AHP amplitude (Baseline: 16.59 ± 0.73 mV; 90 μM: 17.50 ± 1.36 mV; *n* = 8; Two‐tailed paired *t*‐test, *t*(7) = 0.6661, *p* = 0.5267; Figure 4C2) were not affected.

### 3.4 DMT Increased the Intracellular Calcium in Both Females and Males

**FIGURE 5:**
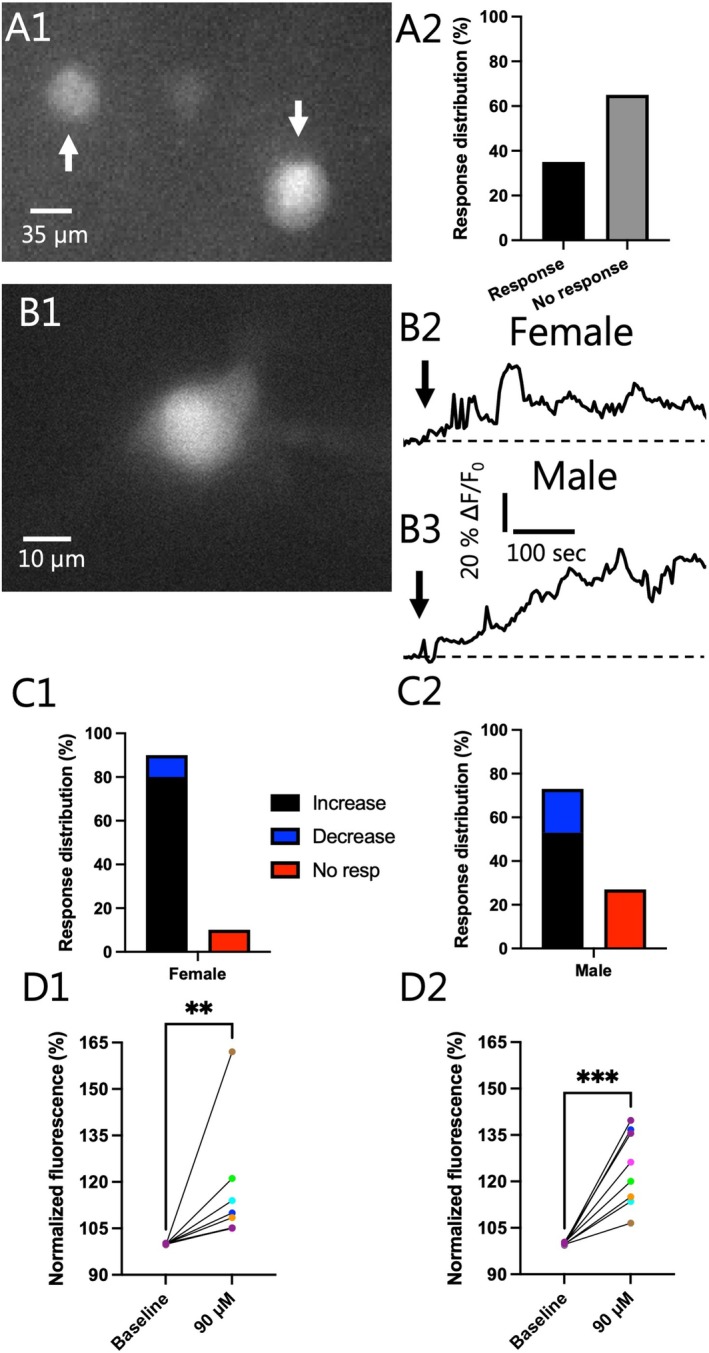
90 μM dimethyltryptamine (DMT) induced changes in calcium in cells in the ventral tegmental area (VTA), and alterations in calcium were noted in the majority of I_h_‐negative VTA neurons. (A1) VTA cells loaded with the cell permeant, Fura‐2‐AM are shown with white arrows under 40× magnification and illuminated at 380 nm wavelength light. (A2) Approximately 1/3 of cells responded with changes in calcium as shown in stacked charts with the black bar indicating the cells that responded (35%) and the gray bar indicating the non‐responsive cells (65%). (B1) A fluorescent image (380 nm) of an I_h_‐negative neuron loaded with Fura‐2‐penta through the patch pipette is shown at 40× magnification. Representative recordings of ΔF/F_0_ over time from a female (B2) and male (B3) I_h_‐negative neuron showing the increase in fluorescence induced by 90 μM DMT. (C1) Stacked bar charts showing the proportion of cells in females that responded with rises in fluorescence (black) and decreases in fluorescence (blue) and the proportion that failed to respond (red), indicating that the majority of cells responded (90%, *n* = 9/10). An increase in ΔF/F_0_ was the most common response (88.9% *n* = 8/9). (C2) Similarly, in males the majority of patched I_h_‐negative VTA neurons responded (73.3%, *n* = 11/15), with the most common response being an increase in fluorescence (72.7%, *n* = 8/11). (D1) Across the female population of cells that responded with an increase in ΔF/F_0_ to 90 μM DMT, the rise was significantly different when normalized data were compared (Baseline: 100.0% ± 0.07%; 90 μM: 116.4% ± 6.8%; Two‐tailed Wilcoxon test, *W* = 36.00, *p* = 0.0078; *n* = 8). (D2) Similarly, the average normalized fluorescence at baseline in male I_h_‐negative neurons was 99.90% ± 0.13%, which significantly increased to 124.1% ± 4.3% upon 90 μM DMT application (Two‐tailed paired *t*‐test, *t*(7) = 5.594, *p* = 0.0008; *n* = 8). “*n*” denotes the number of neurons recorded from and error bars represents mean ± SEM. Asterisks in figures indicates differing alpha levels, *: *p* < 0.0500, **: *p* < 0.0100, ***: *p* < 0.0010, ****: *p* < 0.0001. The data obtained from cell(s) in the same brain slice are shown with the same color in D1, D2.

Our final studies were to evaluate whether DMT could alter intracellular calcium since DMT has been shown to promote structural and functional plasticity, effects that are often mediated by processes known to involve calcium. An initial screening was conducted using calcium imaging in multiple VTA cells (Figure 5A1) to investigate if changes in ΔF/F_0_ could be observed following exposure to 90 μM DMT. In the tested cells, 35% (*n* = 12/34) responded with an alteration in fluorescence suggesting an action on intracellular calcium, whereas in the remaining 65% (*n* = 22/34) we failed to observe a change in fluorescence (Figure 5A2). Notably, the proportion of cells that exhibited transient changes in calcium closely mirrored the known distribution of GABAergic neurons in the VTA, suggesting a potential cell‐type‐specific response. We then used fluorescence‐based single cell calcium imaging in VTA I_h_‐negative neurons (Figure 5B1), which allowed for phenotypic identification. In the majority of VTA I_h_‐negative neurons in females and males, 90 μM DMT increased fluorescence (Figure 5B2,B3), indicative of a rise in intracellular calcium. In females, 90.0% (*n* = 9/10) responded with an alteration in fluorescence when applying 90 μM DMT. In the responding cells, 88.9% (*n* = 8/9) showed an increase in fluorescence (Figure 5C1). Similarly, in males (Figure 5C2), the majority of responding cells (73.3%; *n* = 11/15) showed a DMT‐induced increase in fluorescence (72.7%, *n* = 8/11). We analyzed the amplitude of the rise in fluorescence in both sexes (Figure 5B2,B3). In females, the average increase was 16.4% ± 6.8% from baseline, which was significant (Two‐tailed Wilcoxon test, *W* = 36.00, *p* = 0.0078; *n* = 8; Figure 5D1). Similarly, in male VTA I_h_‐negative neurons, 90 μM increased fluorescence by 24.1% ± 4.3% from baseline, which was also significant (Two‐tailed paired *t*‐test, *t*(7) = 5.594, *p* = 0.0008; *n* = 8; Figure 5D2). These results suggest that DMT can elevate intracellular calcium in I_h_‐negative VTA neurons in both sexes, independent of changes in membrane electrophysiological properties, suggesting a potentially distinct, calcium‐dependent signaling mechanism contributing to its neuroplastic effects.

## 4 Discussion

We determined that 500 nM and 90 μM of DMT did not alter the holding membrane current, membrane potential, or rheobase in female or male I_h_‐negative VTA neurons when employing voltage‐clamp or current‐clamp protocols in mouse brain slices. Additionally, DMT at either concentration did not induce a change in the interval or amplitude of sEPSCs in males or females, indicating that acutely applied DMT does not affect excitatory synaptic transmission within the time frame evaluated. However, an alteration in the I‐V relationship and conductance was noted in females at −40 and −50 mV with 90 μM. Further, 90 μM DMT increased the firing rate in female VTA I_h_‐negative neurons as we observed a significant increase in the number and frequency of APs with a corresponding lower AP interval; however, this alteration in firing did not appear to be due to changes in the duration or amplitude of the AHP. Effects seen with DMT were sex‐based as similar actions were not seen in the I‐V relationship, conductance, or the number, frequency, or interval of APs in male I_h_‐negative neurons. DMT induced a response in intracellular calcium in 33% of the tested cells when evaluated using multiple cell imaging. In single‐cell imaging in which phenotype could be identified, a significant rise in intracellular calcium was induced by DMT in the majority of I_h_‐negative neurons in both female and male VTA.

Very few electrophysiological studies of DMT exist in the literature, and the majority of studies have focused on the electrophysiological actions of DMT on neurons in the cortex or hippocampus. Therefore, to the best of our knowledge, our study represents the first ex vivo report of cellular and synaptic responses of VTA neurons, and more specifically of I_h_‐negative neurons, to acute applications of DMT. Our findings differ in some respects from those conducted evaluating DMT‐mediated responses in other brain regions and under different paradigms, in which effects are evaluated often hours or days after administering a dose of DMT in vivo. We did not observe any differences in the duration or amplitude of sEPSCs when bath applying 500 nM or 90 μM DMT, which is in contrast to other studies that reported that administration of DMT or a close analog of DMT, 5‐methoxy‐DMT (5‐MeO‐DMT), to mice 1 day prior to euthanization was associated with changes in sEPSCs. Specifically, the frequency and amplitude of sEPSCs in layer V pyramidal neurons in rodent brain slices obtained 24 h after 1 and 10 mg/kg DMT treatment were increased (Ly et al. 2018). In another study, a significantly higher amplitude and frequency of sEPSCs were noted in the hippocampal dentate gyrus when brain slices were recorded 3 days post treatment with 5‐MeO‐DMT (Lima da Cruz et al. 2018). Effects of DMT on plasticity have been further shown by structural studies indicating persistent changes induced by DMT in dendritic growth, spine density, and synaptogenesis in the PFC and hippocampus, with effects also observed in human cortical organoids (Ly et al. 2018; Morales‐Garcia et al. 2020; Dakic et al. 2017). Collectively, these studies demonstrate that DMT and 5‐MeO‐DMT promote synaptic plasticity, implying pre‐ or postsynaptic mechanisms that were not observed in our investigation, potentially due to differences in brain region, neuronal phenotype, method of drug exposure, including duration of exposure, and presence of an intact network. Future studies should be conducted to identify the specific factors required to induce DMT mediated plasticity, to determine in which neural regions plasticity can be induced, and to delineate the cellular and circuit‐level mechanisms underlying changes in synaptic strength.

While we saw no changes suggesting plasticity at excitatory synapses, we did note a change in calcium induced by acutely applied DMT in VTA I_h_‐negative neurons, which is interesting in light of the central role that calcium plays in synaptic plasticity through triggering signaling cascades involved in pre and post‐synaptic alterations in synaptic strength, as well as in dendritic remodeling. While our report is the first to investigate whether DMT induces rises in calcium upon acute application ex vivo, a daily dose of 2 mg/kg DMT for 3 weeks prior to euthanasia in 3 × TG‐AD transgenic mice, a model for Alzheimer's disease, restored dysfunctions noted in mitochondrial and cytosolic calcium in hippocampus and PFC slices. Normalization of calcium was speculated to involve IP_3_ receptors subsequent to agonism of the 5‐HT_2A_ receptor resulting in activation of the PLC intracellular cascade, although a fast‐acting sigma‐1 activation could not be ruled out as these receptors are co‐localized with IP_3_ receptors and can result in calcium efflux (Cheng et al. 2024; Hayashi and Su 2001). Our multiple cell imaging revealed that approximately one‐third of the recorded VTA cells responded with rises in calcium, which corresponds with the proportion of GABAergic neurons in this nucleus (Nair‐Roberts et al. 2008; Swanson 1982). In single cell recordings, we confirmed that calcium rises were induced in the majority of I_h_‐negative neurons in the female and male VTA. We did not determine whether calcium rises were due to an IP_3_ mediated intracellular calcium store release, or whether calcium arose through flux across the membrane; however, while not definitive, our failure to see a change in membrane conductance in both sexes suggests that a change in membrane permeability to calcium was not involved. Future studies should investigate the source of calcium influx induced by DMT and assess whether synaptic plasticity can be triggered by acute exposure but requires a delayed period to become functionally or structurally evident.

In our study, we did not observe that acutely applied DMT induced any change in membrane potential, AHP duration, or amplitude in I_h_‐negative neurons in either female or male VTA. These results suggest that acute exposure to DMT may be insufficient to alter intrinsic membrane properties. However, while longer exposure durations could have induced changes in these properties, our data are aligned with findings that daily exposure to 100 μg of 5‐MeO‐DMT for 21 days failed to induce a change in membrane potential, AHP duration, or amplitude in adult hippocampal dentate gyrus neurons (Lima da Cruz et al. 2018). Thus, DMT may not induce changes in core intrinsic membrane properties underlying the AHP or membrane potential even with prolonged exposure; however, the caveat remains that DMT and 5‐MeO‐DMT are not chemically identical and effects on intrinsic properties could be cell type specific and may depend on the particular neuronal phenotype under investigation.

While DMT did not exert actions on synaptic activity or intrinsic properties, we did note a sex‐based change in firing rate and a sex‐ and concentration‐based difference in the I‐V relationship and conductance induced by DMT in VTA of I_h_‐negative neurons, which represents the first time these actions have been reported. However, changes in firing rate induced by 5‐MeO‐DMT and other psychedelics investigated for their anti‐addiction and anti‐depressant properties have been reported. Increases in the firing rate of hippocampal dentate gyrus granule cells were noted in adult mixed sex mice that received 100 μg of 5‐MeO‐DMT 21 days prior to electrophysiological recordings (Lima da Cruz et al. 2018). The firing rate of cingulate cortex, pre limbic cortex, and infralimbic cortex neurons was decreased following a single dose of psilocybin of 0.3 and 1.0 mg/kg in male rats (Purple et al. 2025). In another study, two different changes in firing rate were induced by lysergic acid diethylamide (LSD) in reticular thalamic GABA neurons in male mice (Inserra et al. 2021). In one population, a decrease in firing rate, including burst firing was noted following administration of 20–160 μg/kg *i.p*. of LSD; whereas, in another population, an increase in firing was induced at 80 and 160 μg/kg doses (Inserra et al. 2021). In contrast, we did not note an effect on firing in I_h_‐negative VTA neurons in male brain slices acutely exposed to 90 μM DMT, however, we did observe a significant increase in the firing rate in female brain slices, which could not be attributed to hormonal alterations as the onset of the estrus cycle normally occurs around PND 35 (Caligioni 2009; Nelson et al. 1982), and our mice were younger than this age. Further this effect was dose specific as lower concentrations did not exert a similar effect. Interestingly, we have previously reported that ibogaine, another psychedelic investigated for its anti‐ addiction and anti‐depressant properties reduces firing in I_h_‐negative neurons selectively in male VTA, and we did not note an effect on conductance of ibogaine in either males or females at any tested potential (Eliasen et al. 2025b). In the present study, the significant increase in neuronal firing at membrane potentials near threshold in females induced by DMT was not observed at more depolarized potentials. Importantly, rheobase remained unchanged, which indicates that DMT did not shift the current threshold for spike initiation. Modulation of voltage‐dependent conductances within this near‐threshold window can substantially increase firing probability without altering rheobase, reflecting a change in neuronal gain rather than a shift in baseline excitability. Such state‐ and voltage‐dependent gain modulation is consistent with established principles of neuromodulator control of membrane conductance (Marder 2012) and specifically with evidence that serotonergic signaling via 5‐HT_2A_ receptors can alter voltage‐dependent ionic conductances (Carr et al. 2002; Avesar and Gulledge 2012; Burstein 2021). While we did not investigate the identity or role of the DMT altered conductance, the increase in membrane conductance selectively in females at −40 and −50 mV, a voltage range close to spike initiation may enhance responsiveness to inputs or increase gain while reducing membrane potential fluctuations, thereby contributing to more stable near‐threshold excitability. In contrast, at higher suprathreshold depolarizations the relative contribution of DMT appeared to be limited, potentially due to channel inactivation, suggesting that DMT's effects are more prominent in modulating near‐threshold dynamics that influence spike initiation, rather than altering neuronal responses once firing is fully engaged.

When taken together, these findings indicate that DMT exerts a concentration‐ and voltage‐dependent modulation of neuronal output. The female‐specific effect implies sex‐dependent modulation of these pathways, and that DMT enhances excitability of VTA I_h_‐negative neurons, which could promote signal propagation in females to a greater extent than in males. Because I_h_‐negative VTA neurons are putatively GABAergic and include local interneurons, this DMT‐induced increase in firing could, in turn, dampen the activity of neighboring dopaminergic neurons, thereby reducing DA excitability within the VTA. Such an effect may contribute to therapeutic outcomes specifically in females given that DMT did not enhance firing in male I_h_‐negative neurons. However, while investigation of potential sex‐based differences in psychedelic treatments is mandated (Shadani et al. 2024), currently it remains unknown whether DMT provides greater clinical benefit in females than in males for management of VTA‐related disorders. These findings, together with prior reports, underscore the complexity of psychedelic‐induced changes in neuronal excitability and channel properties across brain regions, cell types, and experimental conditions. Further, they point to the possibility that duration of exposure, sex, and drug concentration are critical determinants of these effects and that psychedelics that confer anti‐addiction and anti‐depressant properties alter neuronal transmission differentially, which requires explicit evaluation.

Taken together, electrophysiological and calcium changes induced by DMT of I_h_‐negative VTA neurons were modest; however, DMT has been shown to modulate large‐scale brain network dynamics and enhance functional connectivity across distributed circuits involved in cognition, emotion, and reward processing, including cortico‐limbic networks and default‐mode/frontoparietal systems. Functional MRI studies report acute increases in global and region‐specific connectivity during DMT administration, effects that correlate with altered subjective states and engage higher‐order associative and socio‐emotional regions (Soares et al. 2024). Additionally, DMT's engagement of multiple receptor systems (e.g., 5‐HT_2A_ and other monoaminergic targets) and its contributions to neuroplastic processes likely involve distributed signaling pathways that do not manifest solely as changes in VTA firing in isolated neurons (Moliner et al. 2023; Ly et al. 2018; Vargas et al. 2023; Fontanilla et al. 2009; Rickli et al. 2016; Cozzi et al. 2009; Olson 2018; Shao et al. 2021; Jiménez 2023). Thus, the apparent limited impact on VTA neurons in our cellular assay may reflect the context‐dependent nature of actions of DMT, which in vivo engage integrative network mechanisms across diverse brain regions that contribute to its therapeutic effects observed in addiction and other neuropsychiatric conditions.

Our study has several limitations. We did not determine the molecular mechanisms through which DMT exerts sex‐dependent effects on firing, induces sex‐dependent and concentration effects on the I‐V relationship and conductance, or evokes concentration and sex‐independent increases in calcium in I_h_‐negative VTA neurons. DMT acts as a partial agonist at multiple serotonergic receptors, including the 5‐HT_2A_ with a nanomolar affinity (~38–462 nM (Sadzot et al. 1989; Rickli et al. 2016; McKenna et al. 1990; Blough et al. 2014; Kozell et al. 2023)), suggesting that at both concentrations utilized in our study, activity at the 5‐HT_2A_ was induced. At micromolar concentrations suggested to be therapeutic, DMT binds to both Sigma‐1 receptors (~15 μM), which mediate calcium signaling, and neuroplasticity and the Trace amine associated receptor 1 (3.3 μM) (Simmler et al. 2016), which is involved in sensitivity to drugs of abuse, mood dysregulation, and hypofunction of NMDA receptors (Rutigliano et al. 2017). Structural studies have shown persistent changes induced by DMT in dendritic growth, spine density, and synaptogenesis in the PFC and hippocampus involving 5‐HT_2A_, TrkB, Mammalian Target of Rapamycin, and Sigma‐1 receptor pathways, with effects also observed in human cortical organoids through NMDA/AMPA and CaMKII/CREB signaling (Ly et al. 2018; Morales‐Garcia et al. 2020; Dakic et al. 2017). Based on binding affinities, it is possible that the two lowest concentrations used of 500 nM and 10 μM primarily activated the 5‐HT_2A_ receptor, whereas the higher concentration of 90 μM might have activated receptors beyond the 5‐HT_2A_ receptor, suggesting that effects on calcium, firing, and conductance noted in females at 90 μM were not specific to activation of 5‐HT_2A_. Another limitation of our study is that we identified recorded neurons as putatively GABAergic based on lack of I_h_ current as the absence of I_h_ in VTA neurons has been proposed as a potential electrophysiological marker of GABAergic phenotype in mice (Morales and Margolis 2017; Lammel et al. 2008). This approach allowed for rapid identification of putative GABA neurons during ex vivo recordings; however, this criterion has limitations as dopaminergic neurons may also exhibit weak I_h_, although this does not appear to be the case in mouse VTA (Zhang et al. 2010; Chieng et al. 2011; Walsh and Han 2014). Regardless, definitive phenotypic identification of cells should be conducted in future using complementary methods such as immunohistochemistry or genetic labeling. In addition, considerations should also be taken of the functional heterogeneity among different GABA populations and whether effects of DMT in the VTA are different across these populations. While the complex mapping of VTA GABAergic projections is in its infancy, it is clear that VTA GABAergic transmission extends well beyond terminations on local dopaminergic neurons, as most GABA neurons in the VTA have both local and distal connections (Al‐Hasani et al. 2021; Breton et al. 2019; Taylor et al. 2014; Yang et al. 2018; Zhou et al. 2022; Oriol et al. 2025). Additionally, adding to the complexity, VTA GABA neurons have also been shown to release both GABA and glutamate from a single axon terminal, raising the possibility that DMT could alter not only inhibitory signaling from I_h_‐negative VTA neurons but also excitatory output (Root et al. 2014). When taken together, future studies need to be conducted to determine the specific receptor mechanisms mediating actions on firing and calcium, and to clarify the concentration‐ and sex‐dependent roles of serotonergic and non‐serotonergic pathways in DMT‐induced modulation of VTA neuronal activity. In addition, genetic indicators of phenotype and future antero‐ and retrograde studies done specifically in GABA VTA neurons and their target regions could be invaluable in terms of revealing this complexity.

## 5 Conclusion

Clinically, the functional relevance of DMT is increasingly supported by well‐controlled studies. A retrospective survey reported that 69% of 441 respondents self‐identified smoking cessation following DMT use, suggesting potential in addiction treatment (Daldegan‐Bueno et al. 2022). Furthermore, in 2019, following completion of the first randomized, placebo‐controlled trial, a rapid and sustained antidepressant effect in treatment‐resistant individuals was reported after administration of a single dose of DMT (Palhano‐Fontes et al. 2019). Complementing this, a one‐year observational study reported over 60% remission in depressive symptoms among patients following a single dose (van Oorsouw et al. 2022). These robust behavioral effects are likely underpinned by cellular and synaptic changes that modulate neural circuits involved in mood and reward. While effects of DMT on functional connectivity in the cortex and hippocampus have been shown as well as actions in a limited population of nuclei, the cellular effects of DMT observed in our study broaden the scope of psychedelic neurobiology beyond cortical systems and actions seen in the VTA could contribute to shaping the mechanistic framework for understanding its reported therapeutic benefits in humans. Our findings reveal that DMT modulates activity in a subset of VTA neurons in a sex‐ and concentration‐dependent manner, with robust calcium signaling observed across sexes in these cells. These results suggest that DMT influences reward‐related circuitry, which may contribute to reported psychological and behavioral effects. Together, these findings underscore the need for translational research to link the cellular and synaptic effects of DMT with its potential long‐term therapeutic outcomes in humans, taking into account variables such as sex and dose‐dependent responses.

## Author Contributions

**Jannik Nicklas Eliasen:** conceptualization, investigation, formal analysis, visualization, writing – original draft, writing – review and editing, funding acquisition, supervision, data curation. **Helene Påbøl Jacobsen:** investigation, formal analysis, writing – review and editing, data curation. **Kristi A. Kohlmeier:** conceptualization, visualization, writing – original draft, writing – review and editing, funding acquisition, supervision, project administration. **Amir Rezagholizadeh:** investigation, formal analysis, writing – review and editing.

## Funding

This work was supported by the Sino‐Danish Center for Education and Research, University of Chinese Academy Sciences, Beijing, China/Aarhus, Denmark [Grant number: 118930_2020].

## Conflicts of Interest

The authors declare no conflicts of interest.

## Data Availability

The data that support the findings of this study are available from the corresponding author upon reasonable request.
